# Selective Recognition of 5-Hydroxytryptamine and Dopamine on a Multi-Walled Carbon Nanotube-Chitosan Hybrid Film-Modified Microelectrode Array

**DOI:** 10.3390/s150101008

**Published:** 2015-01-08

**Authors:** Huiren Xu, Li Wang, Jinping Luo, Yilin Song, Juntao Liu, Song Zhang, Xinxia Cai

**Affiliations:** 1 State Key Laboratory of Transducer Technology, Institute of Electronics Chinese Academy of Sciences, Beijing 100190, China; E-Mails: xuhuiren_2012@163.com (H.X.); wangli2011iecas@163.com (L.W.); jpluo@mail.ie.ac.cn (J.L.); ylsong@mail.ie.ac.cn (Y.S.); liujuntao@mail.ie.ac.cn (J.L.); zhangsong0112@126.com (S.Z.); 2 University of Chinese Academy of Sciences, Beijing 100190, China

**Keywords:** microelectrode array, chitosan, multi-walled nanotubes, dopamine, 5-hydroxytryptamine

## Abstract

It is difficult to determine dopamine (DA) and 5-hydroxytryptamine (5-HT) accurately because of the interference of ascorbic acid (AA) *in vitro*, which has a high concentration and can be oxidized at a potential close to DA and 5-HT at a conventional electrode, combined with the overlapping voltammetric signal of DA and 5-HT at a bare electrode. Herein, chitosan (CS) was used as a stabilizing matrix by electrochemical reaction, and multi-walled carbon nanotubes (MWCNTs) were modified onto the microelectrode array (MEA). The CS-MWCNT hybrid film-modified MEA was quite effective at simultaneously recognizing these species in a mixture and resolved the overlapping anodic peaks of AA, DA and 5-HT into three well-defined oxidation peaks in differential pulse voltammetry (DPV) at −80 mV, 105 mV and 300 mV (*versus* Ag|AgCl), respectively. The linear responses were obtained in the range of 5 × 10^−6^ M to 2 × 10^−4^ M for DA (r = 0.996) and in the range of 1 × 10^−5^ M to 3 × 10^−4^ M for 5-HT (r = 0.999) using the DPV under the presence of a single substance. While DA coexisted with 5-HT in the interference of 3 × 10^−4^ M AA, the linear responses were obtained in the range of 1 × 10^−5^ M to 3 × 10^−4^ M for selective molecular recognition of DA (r = 0.997) and 5-HT (r = 0.997) using the DPV. Therefore, this proposed MEA was successfully used for selective molecular recognition and determination of DA and 5-HT using the DPV, which has a potential application for real-time determination *in vitro* experiments.

## 1. Introduction

Neurotransmitters play an important role in the transmission of the electrical signal from one neuron to the next one. They are chemicals released from the synapses, such as dopamine (DA) and serotonin (5-hydroxytryptamine (5-HT)), which are important catecholamine neurotransmitters in biological systems. Some neurological disorders, such as schizophrenia and Parkinson's disease, result from deficiency of DA, which is widely distributed in the brain tissues for neurochemical message transfer in the mammalian central nervous system. 5-HT is also involved in the brain and plays a crucial role in the emotional system together with other neurotransmitters [1]. Deficiency of 5-HT leads to mental disorders, such as Alzheimer's disease, infantile autism, mental retardation, sleep disorders and depression [2]. Additionally, numerous reports demonstrated that DA and 5-HT affect each other in their respective releasing and play a regulatory role together on the nervous system [3–5]. Selective recognition of 5-hydroxytryptamine and dopamine is meaningful for the detection of neural information. Some major obstacles were encountered in the selective recognition of DA and 5-HT. One is the co-existence of a high concentration of ascorbic acid (AA), which can be oxidized at a potential close to DA and 5-HT at conventional electrodes, and the other is an overlapping voltammetric signal of DA and 5-HT at a bare electrode. Electrochemical techniques based on various approaches have been employed to overcome these difficulties for the determination of DA and 5-HT [6–14].

With the development of micro-electro mechanical system (MEMS) technology, microelectrode arrays (MEAs) have now become a reliable interfacing technique capable of establishing a bidirectional communication between a population of connected neurons and the external world [15]. The microelectrodes have low impedance (lower than 1 M at 1 kHz) with a good cellular sealing [16] and a high charge injection capacity for stimulation [17]. The functional characteristics of the MEA allow analysis of neural function at both the single cell and network level. Additionally, MEAs in different forms permit stable long-term and real-time recordings from cultures and isolated tissues. Compared with conventional electrodes, there are additional challenges for MEAs to achieve selective recognition of DA and 5-HT. On the one hand, a microelectrode array requires modifying the materials directly on a single microelectrode without covering the other microelectrodes. On the other hand, the cyclic voltammetry response of the microelectrodes exhibits the steady or quasi-steady state of the S-shaped curve without an obvious oxidation peak.

Multi-walled carbon nanotubes (MWCNTs) have stimulated increasing interest in the fabrication of supramolecular nano-bio-assemblies and nano-biosensors [18–24] due to their unique combination of excellent stiffness, high tensile strength, high surface area, high chemical stability and high electrical conductivity [25]. One distinct advantage of the carbon nanotubes-systems results from a high effective surface area, which is beneficial not only for enhancing electrochemical currents of diffusing electroactive species, but also for allowing the load of high density electrochemically active (bio-) molecules, which can improve the electrochemical response [26–30]. Furthermore, carbon nanotubes (CNTs) with their nano-size can be widely manipulated to design novel electrode architectures [31,32]. With the advantages of electrocatalytic and electrical conductivity, MWCNTs were selected as the main catalytic material.

Chitosan (CS) polymers are natural amino polysaccharides with abundant amino groups and exhibit good biocompatibility [33]. CS polymers are a very suitable matrix for immobilizing bioactive molecules and constructing biosensors, due to its solubility in slightly acidic solution and from its insolubility in solution with a pH over its pKa [34]. Meanwhile, because of its low fluidness, CS can become locally insoluble once the pH at every site is over its pKa [35]. These facts have encouraged researchers to develop different biosensors utilizing CS as a matrix to detect ethanol [36], NH4^+^ [37], hemoglobin [38], phenol [39], cholesterol [40], choline [41], lactate [42,43] and glucose [44,45]. In addition, the positive attributes of excellent biocompatibility and low toxicity with versatile biological activities, such as antimicrobial activity and low immunogenicity, have provided ample opportunities for real-time detection in *in vitro* and *in vivo* experiments.

Herein, this paper depicts a novel method to modify CS-MWCNT hybrid films directionally onto a single microelectrode by electrochemical deposition. The CS-MWCNT hybrid film-modified MEA is designed for selective recognition of 5-hydroxytryptamine and dopamine using cyclic voltammetry (CV) and differential pulse voltammetry (DPV).

## 2. Experimental Section

### 2.1. Apparatus and Reagents

Cyclic voltammetry (CV) and differential pulse voltammetry (DPV) were performed on a Gamry electrochemical workstation (Gamry Reference 600, Gamry Instruments, Warminster, PA, USA). A Dell E5400 notebook PC was used to collect electrochemical data. Water was purified through a Michem ultrapure water apparatus (Michem, Chengdu, China, resistivity 18 MΩ·cm). Multi-walled carbon nanotubes were purchased (purity >95 wt%) from Xianfeng nanomaterials company (Nanjing, China). The phosphate-buffered saline (PBS, 0.1 M Na_2_HPO_4_-NaH_2_PO_4_-KCl, pH 7.4) was prepared from a PBS tablet (Sigma, St.louis, MO, USA). Dopamine (DA, ≥99%) was purchased from Acros Organics. Serotonin (5-HT, 99%) and ascorbic acid (AA, 99%) were purchased from Alfa Aesar. Chitosan (CS, from crab shells, minimum 85% deacetylated) was purchased from Sigma-Aldrich Co., Ltd (St.louis, MO, USA). All other chemicals were of analytical reagent grade and used without further purification. All experiments were carried out at ambient temperature. The SEM images of the electrode surfaces were produced with scanning electron microscopy (S-3500, Hitachi, Tokyo, Japan).

### 2.2. Preparation of MEA

**Figure 1.**
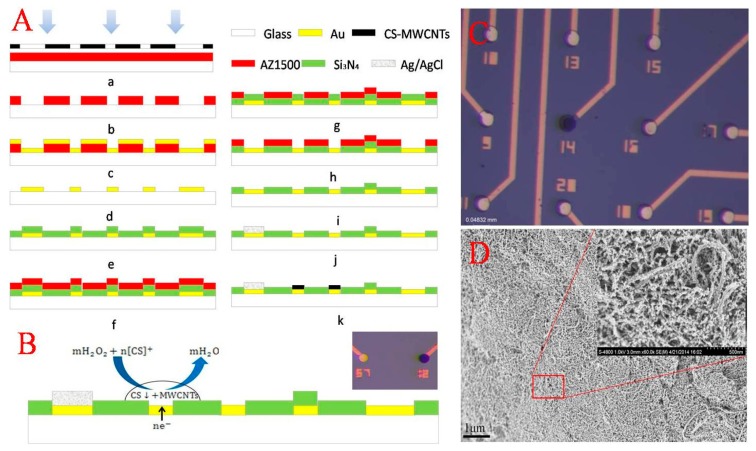
Fabrication and modification of the microelectrode array. (**A**) Microelectrode array (MEA) fabrication process schematic; (**B**) reaction principle of electro-deposition; (**C**) image of the modified microelectrode and bare Au electrode; (**D**) SEM images of the chitosan (CS)-MWCNT hybrid film.

The neural MEA manufactured using standard lithographic processes consists of an Au/Cr conductive layer and a silicon nitride (Si_3_N_4_) insulating layer, as shown in Figure 1A. In brief, a standard commercialized glass was used as the substrate, cleaned in turn by acetone, ethanol and deionized (DI) water, and then, an Au/Cr film (200/30 nm) conductive layer was deposited after the first photolithographic step. The pattern of 60 circular microelectrodes with a diameter of 20 μm and a spacing of 100 μm was presented followed by a lift-off process. Subsequently, a Si_3_N_4_ insulating layer (800 nm) was grown on the substrate using plasma-enhanced chemical vapor deposition before the microelectrode sites were etched in an SF_6_ deep reactive ion etcher (SF_6_ DRIE) for 10 min at a power of 100 W, and the sites of the microelectrodes were displayed. Finally, screen printing was used for the preparation of the reference electrode Ag|AgCl.

### 2.3. Fabrication of CS-MWCNT Hybrid Film

Prior to the experiment, the MEA was reactive-ion etched by oxygen for cleaning. Electrochemical deposition of CS-MWCNT hybrid film was carried out in CS (1.0 wt%) solution containing MWCNTs and H_2_O_2_ (3 wt%) after being ultrasonicated for 30 min. A three-electrode system was used. The counter electrode was Pt, and the reference electrode was Ag|AgCl. The working potential was −0.4 V for 2 min.

The reaction principle is as follows (as shown in Figure 1B): (1) chitosan protonated: CS(Solid) + nH^+^ → [CS]^+^; and (2) electrochemical reaction and electro-deposition of chitosan deprotonated: mH_2_O_2_ + n[CS]^+^ + ne^−^ → CS ↓ +mH_2_O. Because of its low fluidness, CS formed a stabilizing matrix in the alkaline micro-area close to the working electrode by removal of local H^+^ and immobilized the MWCNTs onto the microelectrode surface. By this method, directional modification of the single microelectrode without covering other microelectrodes (as shown in Figure 1C) was achieved.

The oxidation products of DA and 5-HT adsorbed on the electrode surface poison the electrode, resulting the reduction of the response of DA and 5-HT. Performance degradation over time is a challenge for irreversibly-modified methods. With reversibility and simplicity, the reaction principle of CS film has an advantage in reusing MEA, and it is easy to obtain a fresh electrode surface without adsorption by dissolving the hybrid film and re-deposition.

### 2.4. Electrochemical Measurements

Cyclic voltammetry (CV) and differential pulse voltammetry (DPV) were performed on the Gamry workstation connected with a three-electrode system. The CS-MWCNT hybrid film-modified microelectrode as the working electrode, Ag|AgCl as the reference electrode and a platinum wire as the counter electrode were used. DPV experiments were conducted under the instrumental conditions of a 5-mV step size, 25-mV pulse size, 100-ms pulse time and an accumulation time of 400 s.

## 3. Results and Discussion

### 3.1. Characterization of CS-MWCNT Hybrid Film

Figure 1D presents the surface morphology of CS-MWCNT hybrid film, clearly showing the nano-porous structure, which increases the surface area of the microelectrode and reduces the impedance. The nano-porous structure is beneficial for the diffusion of neurotransmitters. In addition, it was observed that there was a large number of MWCNTs exposed on the surface of the CS-MWCNT hybrid film. The advantage of the nano-porous structure with MWCNTs exposed on the surface is similar to the other CNTs systems, resulting in a high effective surface area, which is beneficial, not only for enhancing the electrochemical currents of diffusing electroactive species, but also for allowing a high density load of electrochemically active (bio-) molecules, which can improve the electrochemical signals [26–30].

### 3.2. Electrocatalytic Oxidation of DA and 5-HT

**Figure 2.**
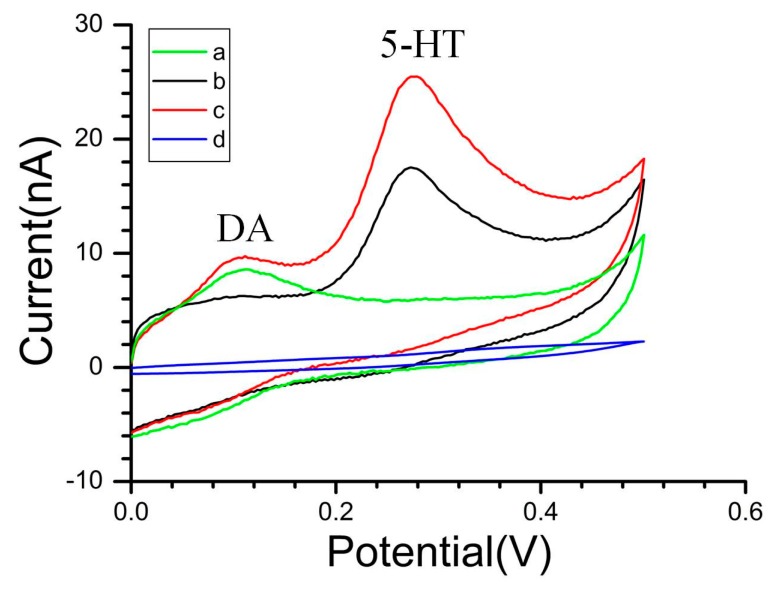
Cyclic voltammetry (CV) of dopamine (DA) and 5-hydroxytryptamine (5-HT) in pH 7.4 phosphate buffer, on CS-MWCNT hybrid film-modified MEA with 50 μM DA (a), 100 μM 5-HT (b), 50 μM DA and 100 μM 5-HT (c), on a bare Au electrode with 50 μM DA and 50 μM 5-HT (d). Scan rate: 50 mV·s^−1^.

As illustrated in Figure 2, the presence of CS-MWCNT hybrid film resulted in the overlapped voltammetric peak being resolved into two well-defined CV peaks (Curve c) at about 103 mV and 273 mV for DA and 5-HT, respectively. Additionally, the oxidation response current of CS-MWCNT hybrid film was higher than the bare Au electrode (Curve d). The oxidation peak separation △Delta;Ep reached 170 mV. This indicates that the CS-MWCNT-modified electrode has better selectivity and response characteristics for the recognition of DA and 5-HT. As was mentioned before, this stable CS-MWCNT hybrid film has a nano-porous structure (Figure 1D). On the one hand, this structure is equivalent to reducing the electrode impedance by increasing the surface area of the electrode and improving the current response; on the other hand, the introduction of MWCNTs means that the CS-MWCNT hybrid film has a wealth of active edge planes of MWCNTs, which improve the catalytic activity of the microelectrode. The catalytic activity of CS-MWCNT hybrid film resulted in oxidation peak separation of the electro-active neurotransmitters and improved the current response.

**Figure 3.**
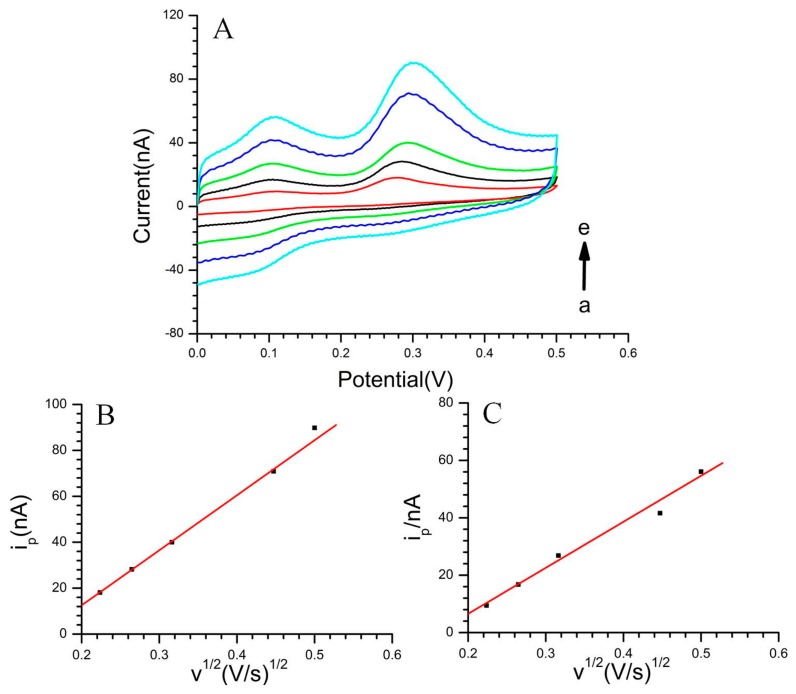
(**A**) CV of CS-MWCNT hybrid film-modified MEA in pH 7.4 phosphate buffer with 50 μM DA and 100 μM 5-HT. Scan rate (a–e): 50 mV·s^−1^, 70 mV·s^−1^, 100 mV·s^−1^, 200 mV·s^−1^, 250 mV·s^−1^; (**B**) Linear relationship between the oxidation peak current of DA with ν^1/2^; (**C**) Linear relationship between the oxidation peak current of 5-HT with ν^1/2^.

As shown in Figure 3, the results of the linear relationship between the oxidation peak current (i_pa_) with the square root of the scan rate (ν^1/2^) indicated that the electrochemical process of DA and 5-HT was diffusion controlled on the CS-MWCNT hybrid film-modified microelectrode. The linear correlation coefficients were r (DA) = 0.9917 (Figure 3B) and r (5-HT) = 0.9992 (Figure 3C), respectively. Therefore, the electrochemical process of the electro-active neurotransmitters is: firstly, the competitive diffusion in the solution and CS-MWCNT hybrid film; then, when the neurotransmitters contact the active edge planes of the MWCNTs, electron transfer occurs; finally, the microelectrode generates a current in response to the occurrence of the electron transfer.

In the process of competitive diffusion, the main mechanism may be the stereo-hindrance effect. Molecules with a larger size and more branched chains need to overcome greater steric hindrance.

**Figure 4.**
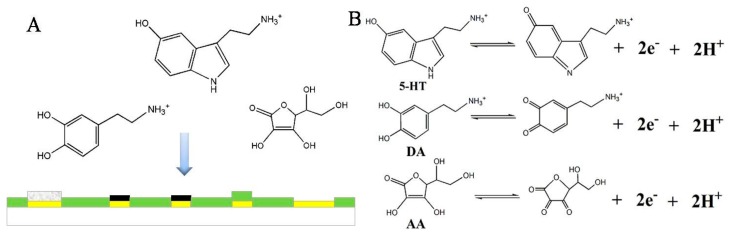
The electrochemical process of DA, 5-HT and ascorbic acid (AA). (**A**) Competitive diffusion in the solution and the CS-MWCNT hybrid film; (**B**) The principle of the oxidation reaction.

The electrocatalytic effect of MWCNTs plays an important role in the process of electron transfer. In addition, edge defect sites or unsatisfied valence on the edge plane of MWCNTs were the active sites. When neurotransmitters contact the active sites on the edge plane of MWCNTs, edge defect sites or unsatisfied valence can play an important role in electrocatalytic processes by enhancing the electron transfer kinetics. The overlap of electron clouds between the molecular and the active sites of MWCNTs resulted in an electronic conjugation effect, reducing the activation energy of the electron transfer. According to previous studies by many researchers [46], the oxidation reactions are shown in Figure 4B.

As shown in Figure 2, the oxidation peak potential of DA shifted more negatively than the oxidation peak potential of 5-HT, about 170 mV, indicating that DA was more prone to oxidation. This result could be due to the stereo-hindrance effect. The molecular size of 5-HT is larger than DA, and the steric hindrance of DA is smaller than 5-HT. As such, DA is more conducive to competitive diffusion in the solution and CS-MWCNT hybrid film.

Compared with the bare Au electrode (Figure 2), the oxidation peak potential of DA and 5-HT on the CS-MWCNT hybrid film-modified electrode shifted negatively and the oxidation current increased. These changes could be due to electrocatalytic effect of the MWCNTs. Due to the similarity of the benzene ring structure, the exposed electron clouds of the active sites of MWCNTs are prone to overlap the electron cloud of DA and 5-HT, resulting in π-π electronic conjugation. The π-π electronic conjugation reduces the activation energy of the electron transfer. Therefore, electron transfer is more likely to occur on the CS-MWCNT hybrid film-modified microelectrode. Additionally, the π-π electronic conjugation resulted in a more negative oxidation peak potential and higher oxidation current than the bare Au electrode.

### 3.3. DPV Response of DA and 5-HT

**Figure 5.**
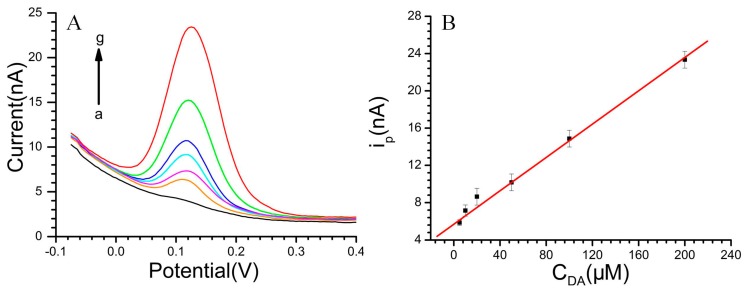
(**A**) DPVs of DA, DA concentration (a–g): 0 to 2 × 10^−4^ M; (**B**) The linear relationship between the DA concentration with the oxidation peak current. Solution: pH 7.4 phosphate buffer.

**Figure 6.**
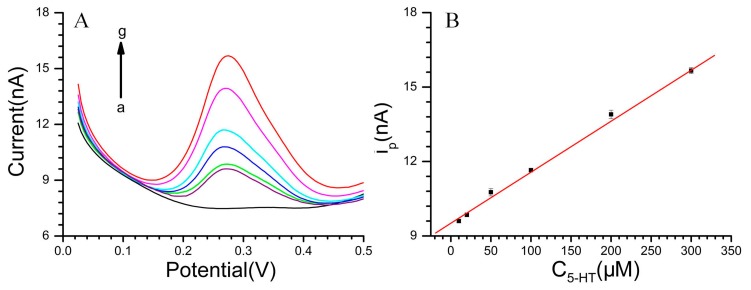
(**A**) DPVs of 5-HT, 5-HT concentration (a–g): 0 to 3 × 10^−4^ M; (**B**) The linear relationship between the 5-HT concentration with the oxidation peak current. Solution: pH 7.4 phosphate buffer.

The DPVs for DA at various concentrations in pH 7.4 PBS and the linear relationship between the DA concentration with the oxidation peak current (i_pa_) are displayed in Figure 5. Additionally, the linear response was obtained between the peak current and DA concentration: i_pa_ (nA) = 5.69145 + 0.08945 C_DA_ (μM) (r = 0.996) in the range of 5 × 10^−6^ M to 2 × 10^−4^ M. The diameter of the microelectrode is 20 μm. The DA sensitivity of the CS-MWCNT hybrid film-modified MEA reached 28.47 mA·mM^−1^·cm^−2^. With regard to 5-HT (Figure 6), the linear range was from 5 × 10^−6^ M to 3 × 10^−4^ M with the equation: i_pa_ (nA) = 9.51295 + 0.02055 C_5-HT_ (μM) (r = 0.999). The 5-HT sensitivity of the CS-MWCNT hybrid film-modified MEA was as high as 6541 μA·mM^−1^·cm^−2^.

### 3.4. Effects of Interferences on the Selective Recognition of DA and 5-HT

In biological environments, the concentration of AA is higher compared to monoamine neurotransmitters and can be oxidized at a potential close to that of DA and 5-HT at the bare electrode. Therefore, it is important to investigate the electrochemical response of DA and 5-HT in the presence of AA.

**Figure 7.**
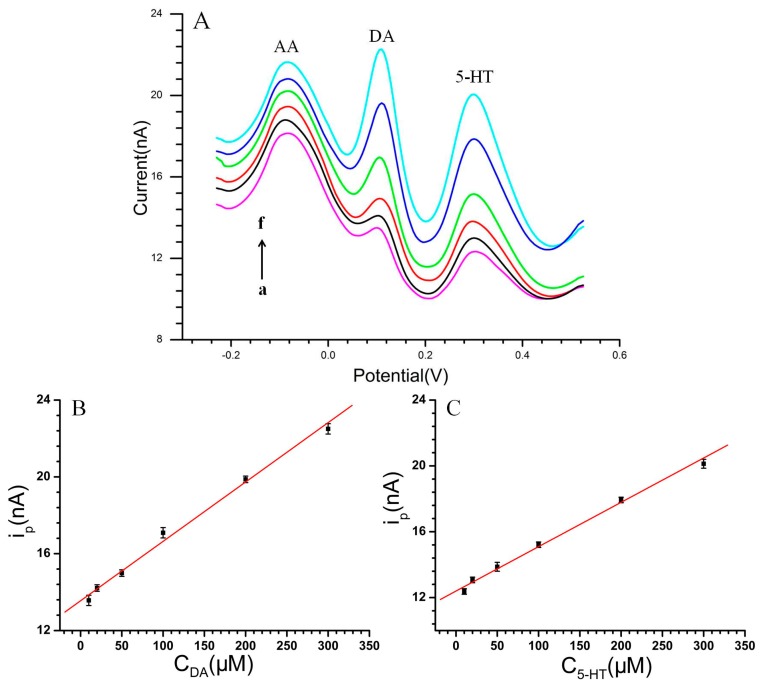
(**A**) DPVs of DA coexisted with 5-HT in the presence of 3 × 10^−3^ M AA, DA and 5-HT concentration (a–f): 1 × 10^−5^ M to 3 × 10^−4^ M; (**B**) The linear relationship between the DA concentration with the oxidation peak current; (**C**) The linear relationship between the 5-HT concentration with the oxidation peak current, respectively. Solution: pH 7.4 phosphate buffer.

The DPV techniques were used to investigate the interferences of 3 × 10^−4^ M AA on DA and 5-HT in pH 7.4 phosphate buffer, as shown in Figure 7A. Under this condition, three separated oxidation peaks were observed at about −80 mV, 105 mV and 300 mV, corresponding to AA, DA and 5-HT, respectively. This indicated that CS-MWCNT hybrid film-modified MEA could achieve the selective recognition of DA and 5-HT in the interference of AA.

The presence of AA resulted in the oxidation peak potential of DA and 5-HT shifting in the positive direction. This may be attributed to the stereo-hindrance effect. The steric hindrance of molecular diffusion increased with the increase of the total concentration of the solution. Therefore, the oxidation overpotential increased and the oxidation peak potential shifted positively. The separation of the peaks between either two peak potentials was large enough to recognize DA and 5-HT individually. The oxidation peak potential of AA was lower than DA and 5-HT, about 185 mV and 380 mV, respectively. This may be due to the heterocyclic structure of AA easily forming the p-π conjugate with the active sites of MWCNTs. The p-π conjugate effect reduced the activation energy of the electron transfer, resulting in a lower overpotential. Furthermore, the molecular size of AA is smaller and is more conductive to competitive diffusion. The p-π conjugate effect and stereo-hindrance effect resulted in a more negative oxidation peak potential of AA.

It can be seen that the peak current of DA increased with the increase of DA concentration. A linear response was obtained between the peak current (i_pa_) and DA concentration (Figure 7B): i_pa_ (nA) = 13.5512 + 0.03092 C_DA_ (μM) (r = 0.997) in the range of 1 × 10^−5^ M to 3 × 10^−4^ M. Figure 7A shows the DPVs of various concentrations of 5-HT at the CS-MWCNT-modified microelectrodes in the presence of AA. The peak height of 5-HT increased with increasing 5-HT concentration. With regard to 5-HT, the linear range was from 1 × 10^−5^ M to 3 × 10^−4^ M with the equation: i_pa_ (nA) = 12.39908 + 0.02695 C_5-HT_ (μM) (r = 0.997) (as shown in Figure 7C).

While the electro-active neurotransmitters coexisted with 3 × 10^−4^ M AA, the sensitivity of the CS-MWCNT hybrid film-modified MEA was as high as 9842 μA·mM^−1^·cm^−2^ and 8578 μA·mM^−1^·cm^−2^ for determination of DA and 5-HT, respectively. This indicated that CS-MWCNT hybrid film-modified MEA could be used for the selective determination of DA and 5-HT in the interference of AA.

In order to characterize the reproducibility of the modified microelectrode, repetitive measurements were carried out. MEA was reactive-ion etched by oxygen for cleaning and underwent successive cyclic sweeping between 0 V and 0.5 V in the blank PBS to give a fresh microelectrode surface (five cycles). This cleaning step allowed the oxidation products to move away from the microelectrode surface, and so, a fresh surface was attained. After that, the MEA was used for another measurement with the same conditions. The relative standard deviation (RSD) was about 3.2% for five successive determinations, indicating that the designed sensor had excellent reproducibility.

## 4. Conclusions

This work demonstrated that CS-MWCNT hybrid film exhibited remarkably electrocatalytic activity toward DA and 5-HT oxidation, improving the response currents and lowering their oxidation overpotentials, which was attributed to the synergic effect of CS and MWCNTs. The nano-porous structure of CS-MWCNT hybrid film reduced the impedance and increased the mass transportation rate of these reactions. The electrocatalytic effect of MWCNTs reduced the activation energy of the electron transfer and increased the electronic conductivity.

In addition, the excellent electrocatalytic activity of the CS-MWCNT hybrid film can resolve the overlapping anodic peaks of DA and 5-HT into two well-defined peaks at 103 mV and 273 mV with a separation of about 170 mV in CV, and three separated oxidation peaks were observed at −80 mV, 105 mV, 300 mV, corresponding to AA, DA and 5-HT, respectively, in DPV. The CS-MWCNT hybrid film-modified MEA was applied successfully for the selective recognition and determination of 5-HT and DA. While DA coexisted with 5-HT in the interference of AA, the CS-MWCNT hybrid film-modified MEA can measure micromolar levels of DA and 5-HT in linear ranges of 1 × 10^−5^ M to 3 × 10^−4^ M, with a good linear correlation, r _DA_= 0.997 and r _5-HT_ = 0.997, respectively. The sensitivity of the CS-MWCNT hybrid film-modified MEA was as high as 9842 μA·mM^−1^·cm^−2^ for DA and 8578 μA·mM^−1^·cm^−2^ for 5-HT.
